# Trends in emotional functioning and psychosocial wellbeing in breast cancer survivors: a prospective cohort study using patient-reported outcome measures

**DOI:** 10.1186/s12905-023-02243-0

**Published:** 2023-03-30

**Authors:** Sri K. Devarakonda, Reinier Timman, Paul F. Bouvy, Arvind Oemrawsingh, Inge Apon, Marc A. M. Mureau, Linetta B. Koppert, Leonieke W. Kranenburg

**Affiliations:** 1 Department of Psychiatry, Section Medical Psychology and Psychotherapy, Erasmus University Medical Center, Dr. Molewaterplein 40, 3015 GD Rotterdam, The Netherlands; 2 Center for Medical Decision Making, Department of Public Health, Erasmus University Medical Center, Rotterdam, the Netherlands; 3 Department of Plastic and Reconstructive Surgery, Erasmus MC Cancer Institute, Erasmus University Medical Center, Rotterdam, The Netherlands; 4 Department of Surgical Oncology, Erasmus MC Cancer Institute, Erasmus University Medical Center, Rotterdam, the Netherlands

**Keywords:** Patient reported outcome measures, EORTC-QLQ-C30, BREAST-Q, Breast cancer, Emotional functioning, Psychosocial wellbeing

## Abstract

**Background:**

A breast cancer diagnosis can threaten every aspect of a woman’s wellbeing, including her mental health. With the growing number of breast cancer survivors, studies addressing mental health in this population are of increasing importance now more than ever. Therefore, the current study investigated trends in emotional functioning and psychosocial wellbeing of breast cancer survivors, and the demographic and treatment characteristics that may influence these trends.

**Methods:**

Prospectively collected data of women treated for breast cancer at the Erasmus MC were analyzed in this study using a cohort study design. Emotional functioning was measured using the EORTC-QLQ-C30, while psychosocial wellbeing was measured using the BREAST-Q. Type of surgery, age, family status and employment status of study participants were retrieved, and multilevel analyses were performed to identify trends in emotional functioning and psychosocial wellbeing and to determine the relationship between aforementioned characteristics and these outcomes.

**Results:**

Three hundred thirty-four cancer survivors were analyzed. Psychosocial wellbeing declined, but emotional functioning showed a steady improvement over time. Women who underwent breast reconstruction showed a steeper increase in their emotional functioning, and women with no partner or children showed a marginal decline in psychosocial wellbeing between baseline and 12 months after surgery.

**Conclusions:**

These findings can be utilized by healthcare teams to identify breast cancer patients at risk for emotional problems and to provide adequate psychological support to those women who need help dealing with their emotions and self-concept in order to optimize clinical treatment.

## Background

Breast cancer is the leading female malignancy and cause of death for women in both developing and developed countries [1]. It is the most common cancer diagnosis in women in the Netherlands [2]. Significant advancements in cancer diagnostics and treatment over the years have rendered the lifetime risk of breast cancer death to drop from 1 out of 22 women in 1990 and 1 out of 24 in 2000 to 1 out of 27 in 2010 [2]. Given this long survival time, studies focusing on quality of life of (long-term) breast cancer survivors more and more become relevant [3]. The reason being that the experience of cancer is known for its effects on mental health. Some studies state that cancer and its treatment can be so traumatizing that the distress associated with cancer has been compared to the course and symptom structure of post-traumatic stress disorder [4–6].

Studies documenting long-term experiences of breast cancer survivors have found that as many as 80% of breast cancer patients experience a considerable amount of distress by virtue of their lifechanging diagnosis and the treatment that follows [7–10]. They found that the pretreatment phase (phase between diagnosis and initial treatment) is marked by the most critical changes to one’s self-concept and lifestyle [11–15]. This is the period where the women might experience increased vulnerability, uncertainty about changes in their corporal identities in addition to existential concerns and rise in symptoms of anxiety and depression [7, 8, 16–19]. Despite all these physical and personal struggles, these women might be expected to get on with their roles, and function like everyone else [18–20]. The construct that reflects the women’s perceived judgments about their behaviors, self-concept, and abilities that are affected by their cancer journey, from here on, will be referred to as *psychosocial wellbeing,* whereas, *emotional functioning* will be understood in relation to the women’s negative emotions, such as irritability and tension [16–25].

While some studies have shown that between 10 and 30% of women experience persistent emotional disturbances, and psychological distress, most studies emphasize the transient nature of dysfunctions in women’s wellbeing: after enduring the distress during the pretreatment phase, the women return (close to) their normal levels of functioning [8–11]. Most studies either focus on the emotional or psychosocial aspects of a breast cancer journey, but we believe that examining the trends in both emotional functioning and psychosocial wellbeing together can give us a fuller picture in identifying potential points in the trajectory of breast cancer where women are most vulnerable to respond to adverse effects of breast cancer (treatment) with diminished levels of functioning, and further to identify demographic and treatment factors that influence these outcomes. Generally speaking, some factors are associated with better wellbeing outcomes, such as having high levels of social support, certain treatments, being older, and being employed [14, 15, 26, 27]. With reference to different surgery types, recent large studies have found better “psychosocial well-being” outcomes for breast reconstruction compared to mastectomy over a 5-year period [28], and for breast conserving surgery with radiation compared to mastectomy and reconstruction without radiation therapy over an on average 10 year period [29]. Although large scale studies offer important information for specific groups of patients, next to large scale data on quality of life outcomes in this group, qualitative studies in this field point toward the importance of understanding the impact of breast cancer on an individual basis, for clinical care to be effective [30].

Therefore, the current study aimed to find out how levels of emotional functioning and psychosocial wellbeing in breast cancer survivors changed as a function of time. Based on the literature, it was expected that patients would follow a U-shaped trend: wellbeing and functioning levels would first decline, then incline as a function of time by virtue of *personal factors*, such as age and employment status, *social factors* such as family support, and *treatment factors* such as type of surgery they underwent. Therefore, from these trends that emerged, the study further aimed to identify factors or characteristics associated with these outcomes, and based on previous literature, these factors were age at the time of diagnosis, family status, type of surgery and employment status [14, 26, 27].

## Methods

The employed cohort study was conducted to explore the outcomes of longitudinally collected patient-reported outcome measures (PROMs). This study was part of an ongoing set of studies investigating the quality of life of breast cancer patients, which was approved by the Medical Ethics Review Committee of the Erasmus University Medical Center (MEC-2018-1015). All data, including baseline patient characteristics and PROMs, were derived from the “Zorgmonitor”. The Zorgmonitor is an institution-specific online database that contains various PROMs collected at different time points [31, 32]. The PROMs are administered to all the breast cancer patients that got diagnosed with breast cancer at Erasmus MC online, and these questionnaires were self-report. PROMs were administered preoperatively (T0), three (T3, within the subset of patient treated with neo-adjuvant systemic therapy) and 6 months (T6) after surgery, a year (T12) postoperatively and yearly thereafter. Since only the participants receiving neoadjuvant chemotherapy were administered the questionnaires at timepoint T3, and since not enough women reached the timepoint T36 after their surgeries, these two timepoints were excluded from the analysis so as to not let small numbers unduly skew the trends. As part of the routine care protocol, informed written consent was obtained from all the patients in this study during the administration of the initial questionnaires at T0 for storing their information in the “Zorgmonitor” and using it for research purposes thereafter. The date of data extraction was March 4th, 2019.

### Cohort demographic and treatment characteristics

All breast cancer patients (≥ 18 years) treated at the Academic Breast Cancer Center, Erasmus MC between October 2015 and March 2019 were included in this study. The demographic characteristics of interest were patient’s age at the time of diagnosis, family status and employment status. Type of surgery was classified as “none” (if they had not yet undergone any surgery), “breast-conserving therapy” (BCT), “mastectomy”, and “reconstruction”. Family status was classified as “no partner/children”, “partner”, “children”, “partner and children”, while employment status was categorized as “employed” and “unemployed”. Age was kept as a continuous variable.

### Patient-reported outcome measures

The PROMs used in this study were the EORTC-QLQ-C30 (The European Organization for Research and Treatment of Cancer Quality of Life Questionnaire) and the BREAST-Q [33, 34]. Respective licenses were obtained for the usage of both these questionnaires for academic purposes by the *Academisch Borstkanker Centrum* (ABC). For this study, the subscales “Emotional Functioning” (EORTC-QLQ-C30) and “Psychosocial Wellbeing” (BREAST-Q) were analyzed. EORTC-QLQ-C30 was administered at timepoints T0, T6, T12, and T24, while BREAST-Q was administered only at T0, T6 and T12.

#### ***EO***RTC-QLQ-C30

This questionnaire measures multiple dimensions of quality of life in cancer patients. It includes 30 questions categorized into five functional scales (physical, social, role, emotional and cognitive), three symptom scales (fatigue, pain and nausea/vomiting), a global health status scale, and additional single items which address common symptoms of cancer patients. The “Emotional Functioning” scale used in this study comprises four questions with respect to the emotional functioning of the respondent in the previous week: “Did you feel tense?”, “Did you worry?”, “Did you feel irritable?”, and “Did you feel depressed?”. Responses are measured on a 4-point Likert scale, and scores are standardized into a range from 0 to 100 with higher scores representing better functioning [35]. EORTC-QLQ-C30 is a strongly validated and reliable measure of QoL which has been translated into many languages [36–38]. The Dutch version of this questionnaire shows high internal consistency (Cronbach’s α = 0.86) for the “Emotional Functioning” scale used in the study [33].

#### BREAST-Q

The BREAST-Q was developed to quantify the effect of different types of breast surgery on quality of life. It consists of six subscales, namely, “Psychosocial Wellbeing”, “Physical Wellbeing”, “Sexual Wellbeing”, “Satisfaction with Breasts”, “Satisfaction with Outcome” and “Satisfaction with Care”. The “Psychosocial Wellbeing” subscale used in this study comprises 10 items for breast conserving therapy (BCT), mastectomy and reconstruction modules with respect to how often the respondent felt the following things about her breasts in the previous 2 weeks: “Felt comfortable in a social situation”, “Felt capable of doing things you want to do”, “Felt emotionally healthy”, “Felt as worthy as other women”, “Felt confident”, “Felt feminine with clothes on”, “Felt that you have accepted your body”, “Felt normal”, “Felt like other women” and “Felt attractive”. Responses are measured on a 5-point Likert scale, and transformed into a range from 0 to 100 with higher values representing a better wellbeing [34]. The preoperative version was administered to all patients at baseline, while surgery-specific versions (breast-conserving therapy, mastectomy or reconstruction) were administered at follow-ups. This questionnaire has a good reliability with Cronbach’s α > 0.80 for all subscales [34, 39, 40]. The “Psychosocial Wellbeing” subscale shows high internal consistencies (Cronbach’s α = 0.95, 0.95, 0.96) for BCT, mastectomy, and reconstruction modules, respectively [40].

### Statistical analyses

Longitudinal analyses were performed with multi-level regression analyses, namely Multilevel Modeling (MLM), for both outcome variables. The covariance structures were determined with restricted maximum likelihood, while the fixed part of the models with ordinary maximum likelihood. ‘Months since baseline’ or ‘m’ was constructed as the difference between each patient’s date of response to questionnaires at T0, T6, T12 and T24, and their registration date. To identify linear and possible non-linear changes in subscale scores, ‘m’ and the logarithm of ‘m’ were entered as fixed covariates. Each demographic and treatment characteristic were then separately entered as a covariate to the previous models, resulting in eight multilevel models.

MLM analysis can utilize time-series data to identify patterns in responses of participants at different timepoints. Because the analysis can model a trend for each participants’ responses, it can accommodate considerably for the missing data in our dataset by analyzing all available data with minimal loss of information, as well as, corrects any biases that may exist as a result of the association between the missing data and any characteristics present in the model [41]. For example, if a participant responded to BREAST-Q only at T0 and T12, the analysis estimates the “rate of change” between those two timepoints for that participant. Since we have a good sample size for cohort studies [42], even though the participant in question did not respond at some other timepoints, the missing values do not unduly influence the final trends, since these are modeled using information from other respondents. This is also why even though BREAST Q is not administered to the participants at T24, an MLM analysis can model and predict what the trends could look like using the information from the other timepoints. It is important to remember though that the trends beyond T12 are merely artefacts predicted by the model and are not based on actual data at T24, but are a close estimation of what the actual trend would look like should we have sufficient data available for T24.

The predictor effects for each demographic and treatment characteristic were determined for subscale scores at T12; which meant that, for example, if we are interested to see how different types of surgery influence the trends in emotional functioning, the predictor effect applies to the trend between T0 and T12 (T12-T0). The reason for choosing T12 for testing predictor effects was because BREAST Q was not administered at T24. Cohen’s d was manually calculated for the outcomes from each of these models. Data was analyzed using IBM Statistical Package for Social Sciences (SPSS) version 24.0 software [43].

## Results

### Sample characteristics

**Fig. 1:**
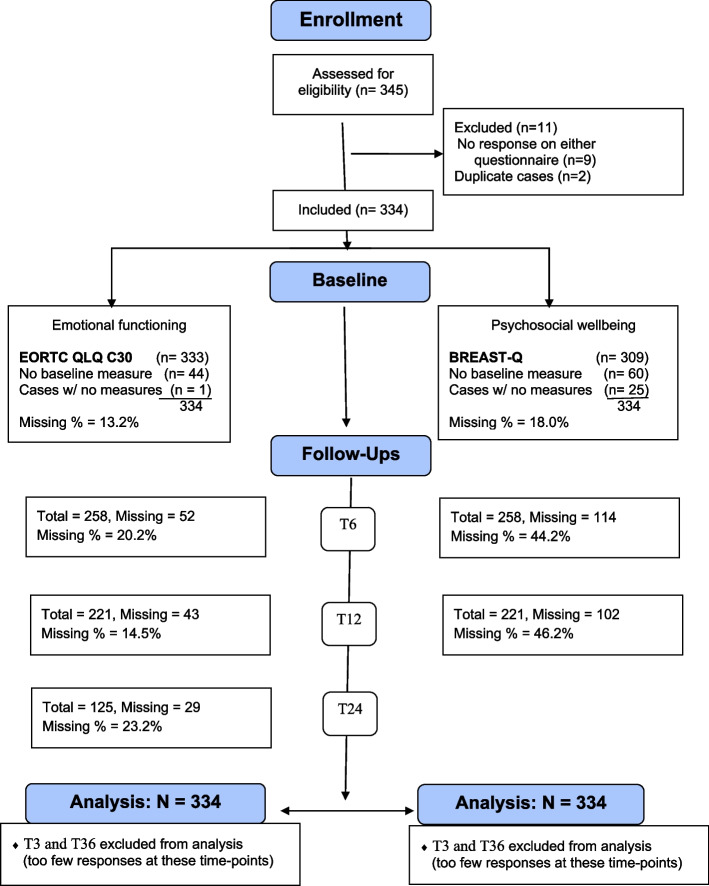
CONSORT flowchart. This flow diagram reports the number of respondents at baseline and follow-ups for EORTC-QLQ-C30’s Emotional Functioning subscale and for BREAST-Q’s Psychosocial Wellbeing subscale

The initial sample size was 345 patients. After data cleaning, participants were excluded if they had not completed the questionnaires at any time point (*N* = 9) or if they were duplicates (*N* = 2). 334 participants were ultimately analyzed. The CONSORT flow chart of dropout rates at each time point is provided in Fig. 1.

Table 1 shows that BCT was the most common surgery undergone by study participants. Most participants in this sample were employed and had both a partner and children. Average age was 52.7 years. However, women in the (mastectomy plus) reconstruction group were 14.6 and 14.5 years younger than those in the mastectomy (without reconstruction) and lumpectomy groups, respectively (*p* <  0.001), while employed women were 15.4 years younger than their counterparts (*p* <  0.001). The 4.2% of women who had not undergone surgery (“none” category) yet, consisted of women who either received neoadjuvant endocrine treatment for a longer time and had not received their surgery yet, or had distant metastases and went on palliative treatment.

**Table 1:** Baseline Demographic characteristics of the sample of breast cancer survivors (*N* = 334)

Predictors	Categories	N *(%)*	Mean age per group (years)	SD
Age			52.74	14.0
Type of surgery	None	14 (4.2)	44.4	14.4
	BCT	188 (56.3)	54.9	13.0
	Mastectomy	92 (27.5)	55.0	14.8
	Breast reconstruction	40 (12.0)	40.4	8.9
Family status	No partner/children	49 (14.7)	55.8	15.2
	Partner	104 (31.1)	55.2	14.6
	Children	15 (4.5)	57.6	11.2
	Partner and children	162 (48.5)	50.0	13.0
Employment status	Unemployed	125 (37.4)	62.5	12.6
	Employed	248 (61.1)	47.0	11.5

*BCT* breast conserving therapy

### Trends in “emotional functioning” and “psychosocial wellbeing”

**Fig. 2:**
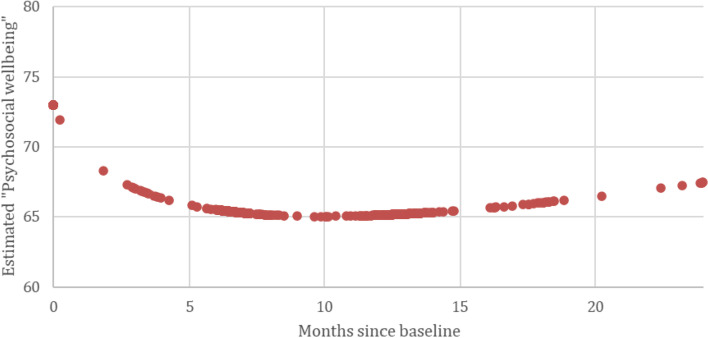
Trends in estimated Psychosocial Wellbeing up to 24 months after the surgery. The data shows that despite the decline in psychosocial wellbeing of breast cancer survivors up to 10–12 months after their surgery, the modelled data suggest that their psychosocial wellbeing may show improvements as a function of time

The changes in emotional functioning and psychosocial wellbeing at different timepoints are presented in Table 2, along with their effect sizes. The estimates of the multilevel analyses are displayed in Appendix. These tables show that emotional functioning and psychosocial wellbeing both had comparable baseline scores (Emotional Functioning: *est. =* 72.99, *SE =* 1.26, *p* <  0.001, Psychosocial Wellbeing: *est. =* 72.98, *SE =* 1.10, *p* <  0.001). The average baseline scores for the Emotional Functioning subscale in our study are close to the reference values for the same subscale in breast cancer population between the ages of 50–59 (*M =* 70.5, *SD* = 22.3) [44], and to the EORTC general population reference value for the subscale (*M =* 76.3, *SD* = 22.8) [45]. However, the normative scores for Psychosocial Wellbeing in breast cancer patients showed a greater range between 57 [46] and 71.3 ± 19.6 [47] depending on the study [48–50], while the general population reference values remained close to the scores found in this study (*M =* 69.5, *SD =* 18.7) [47]. Emotional functioning of breast cancer survivors significantly increased since baseline (log time *est.* = 2.83, *SE* = 0.99, *p* = 0.004), while psychosocial wellbeing significantly declined until a year after surgery (log time *est.* = − 5.37, *SE* = 1.94, *p* = 0.006). However, ‘estimated psychosocial wellbeing’ scores that were modeled based on the analysis (Fig. 2) show that psychosocial wellbeing may show a slight increase followinga year after surgery.

**Table 2:** Estimates for changes in “Emotional Functioning” and “Psychosocial Wellbeing” at different time points

	Time points	Emotional functioning			Psychosocial wellbeing		
		est.	d**	p*	est.	d**	p*
Baseline	T0	72.99			72.98		
Change since baseline	T6	5.28	0.29	< 0.001	−7.51	−0.41	< 0.001
	T12	6.82	0.31	< 0.001	−7.90	− 0.43	< 0.001
	T24	8.23	0.45	< 0.001			
Predictor effects at 12 months							
**Type of surgery**							
BCT		8.22	0.54	< 0.001	−6.93	−0.38	0.001
Mastectomy		1.35	0.09	0.599	−9.03	−0.50	< 0.001
Reconstruction		13.08	0.87	< 0.001	−5.05	−0.28	0.126
Differences between trends in types of surgery at 12 months							
Mastectomy - BCT		−6.87	−0.46	0.025	−2.09	−0.12	0.512
Reconstruction - BCT		4.87	0.32	0.234	1.88	0.10	0.631
Mastectomy - Reconstruction		−11.74	−0.78	0.010	−3.97	−0.22	0.330
**Family status**							
No Partner/Children		3.00	0.14	0.391	−10.34	−0.56	0.008
Partner		6.94	0.31	0.002	−9.04	−0.49	< 0.001
Children		11.81	0.53	0.047	−5.31	−0.29	0.356
Partner & Children		7.28	0.33	< 0.001	−6.50	−0.35	0.002
**Age**							
At mean age		6.54	0.30	< 0.001	−8.01	−0.44	< 0.001
10 years older		4.45	0.20	0.007	−8.54	−0.46	< 0.001
**Employment status**							
Unemployed		5.72	0.26	0.010	−8.69	−0.47	< 0.001
Employed		7.56	0.34	< 0.001	−7.48	−0.41	< 0.001

*p values = sig. of differences of time-point with baseline

**d = Cohen’s d effect size

### Differences between groups, and predictor effects on trends in emotional and psychosocial wellbeing

Table 2 shows the predictor effects on changes in emotional functioning and psychosocial wellbeing between baseline and 12 months (i.e. difference between T12 and T0 for each category of predictor variable). T12 was chosen as the primary timepoint for comparing the groups because psychosocial wellbeing was not recorded at T24.

**Fig. 3:**
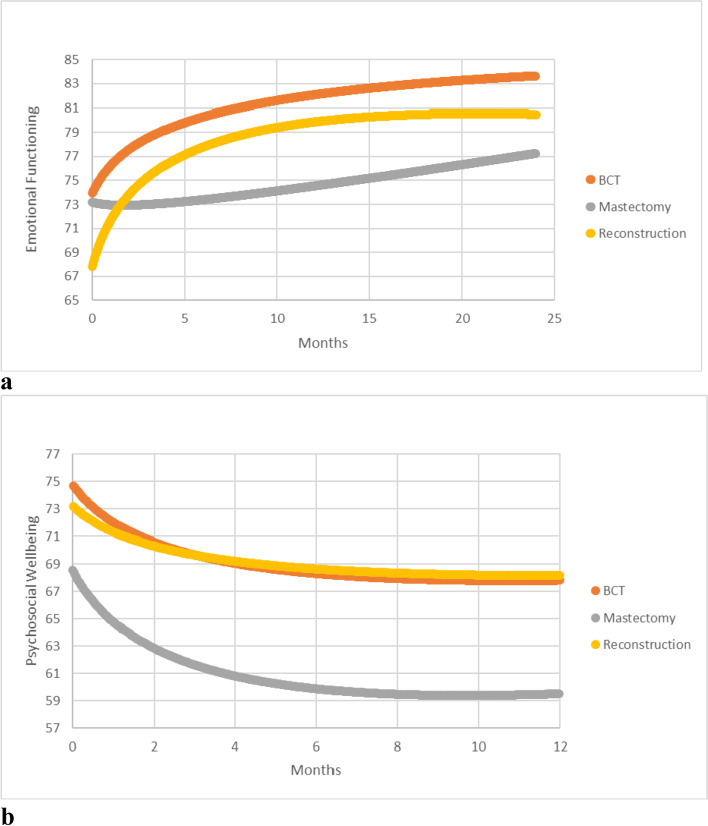
**a** Trends in Emotional Functioning. **b** Trends in Psychosocial Wellbeing. These figures display the different patterns in the emotional functioning and psychosocial wellbeing scores of women based on different surgery types, namely BCT: Breast conservation therapy, mastectomy and breast reconstruction surgery. The data show that while the emotional functioning follows a pattern of increase over time, the psychosocial wellbeing declines over time for all surgery types. Women who underwent mastectomy seem to have the least scores in both their emotional functioning at the end of two years after surgery, and in their psychosocial wellbeing at the end of a year after surgery

Predictor effects of surgery type show that while emotional functioning of the mastectomy group remained fairly stable between baseline and T12 (MAS *est. =* 1.35, *d* = 0.09, *p* = 0.599), BCT and reconstruction groups showed a significant increase in functioning, with reconstruction group showing the sharpest increase (BCT *est. =* 8.22, *d* = 0.54, *p* <  0.001; REC *est. =* 13.08, *d* = 0.87, *p <* 0.001). This means that the emotional functioning of women who underwent reconstruction in our sample seemed to increase faster than those who underwent BCT or mastectomy. Psychosocial wellbeing showed a significant decline between baseline and T12 for BCT and mastectomy groups (BCT *est. =* − 6.93, *d =* − 0.38, *p* = 0.001; MAS *est. =* − 9.03, *d =* − 0.50, *p* <  0.001), but not for reconstruction (REC *est.* = − 5.05, *d* = 0.28, *p* = 0.126). The differences in the trajectories in emotional functioning and psychosocial wellbeing for different surgery types are displayed in Fig. 3.

All family situations except the group with no partner or children showed an incline in emotional functioning between baseline and 12 months after surgery (T12). Psychosocial wellbeing declined for all family situations, but the decline was the greatest for the group with no partner or children. However, neither of these findings were statistically significant. Employment status and age were not significantly related to trends either in emotional functioning or psychosocial wellbeing.

## Discussion

The main goal of this study was to identify whether there were any patterns in emotional functioning and psychosocial wellbeing in breast cancer survivors over time. The results showed that psychosocial wellbeing of our sample decreased over a period of 1 year. Hence, psychosocial wellbeing partly followed the pattern that is in line with findings for the first year in other studies [12, 13] However, emotional functioning followed a different trend as it increased with time. Some studies on post-traumatic stress in breast cancer survivors have considered these two concepts to be very closely related [6, 11, 51, 52], and some others saw these concepts as different, but often influenced by each other [53–57]. While this is not the first study to identify that wellbeing and quality of life are not reflective of one another [22, 57, 58], this study makes a novel contribution to the breast cancer literature by extending this finding to the longitudinal patterns in emotional functioning and (breast-specific) psychosocial wellbeing. These differences between the trajectories of emotional functioning and psychosocial wellbeing can be explained by looking closely at the differences between the emotional functioning subscale of EORTC-QLQ-C30 and the psychosocial wellbeing subscale of BREAST-Q. The emotional functioning subscale measured the general determinants of negative mood, such as anxiety, irritability, and depression [33], whereas the psychosocial wellbeing subscale was more specific, and corresponded to perceptions of self-worth and abilities with respect to the breasts [34].

A cancer diagnosis is marked by fears and uncertainty about treatment, stress, anger and depressive symptoms [7, 8, 59]. This is especially true for women with a history of mental health conditions or preexisting symptoms, which are not factors this study controlled for [60]. This could explain why the emotional functioning in this study was the lowest at baseline and consistently increased with time thereafter, a finding also reported by studies investigating anxiety and depressive symptoms in different types of cancer patients [11, 14, 15, 25, 26]. By contrast, the psychosocial wellbeing was the highest at T0. Considering that this measurement was taken before the participants had undergone any breast surgery, it might explain why psychosocial wellbeing with respect to their breasts is highest preoperatively [34, 39]. The trajectory showed a dip in psychosocial wellbeing at 6 months after surgery, which persisted till 1 year postoperatively. This was in line with findings about the pretreatment phase [11–15], which disrupts one’s self-concept. The baseline scores for both the scales was found to be in-line with the normative data available from previous studies, indicating that our sample started the cancer journey with average emotional functioning and psychosocial wellbeing in comparison to the normative clinical group. Incorporating cancer into one’s life and self-concept is a dynamic process of maintaining mental wellbeing and this data suggests that this process might take more than a year to accomplish. The pattern of adjustment depicted by studies comparing the psychosocial aspects of breast cancer patients with controls show relatively high psychosocial well-being scores for breast cancer patients compared to non-cancer controls with increased scores over time since surgery [46–48, 61]. A possible explanation for this finding is that survivors have found an new equilibrium by incorporating cancer into their lives, and appreciate life more after diagnosis, resulting in higher psychosocial wellbeing scores. Indeed coping, the ability to adjust to changing circumstances, is likely to be of relevance here [62]. Further, beyond just looking at the phrasing of questions within the two questionnaires, since we did not aim to determine (a direct or indirect) relationship between both the outcome variables, any conclusions we draw from the trends would be speculative in nature. Therefore, we believe this finding opens up an avenue for further research to explore longitudinal trends in emotional functioning in conjunction with that of psychosocial wellbeing, and the role of emotional coping on the outcome variables.

One of our other significant findings was that the reconstruction group had both the highest increase in emotional functioning and an insignificant decline in psychosocial wellbeing between baseline and a year after surgery. This contrasts with the findings from many studies, which found that women who received breast reconstruction had more mood disturbances and distress than those who underwent mastectomy, up to twelve months after their surgery [11, 26, 59, 60, 63, 64], but is partially corroborated by some other studies focused on psychosocial wellbeing [28, 46–48, 61]. Several studies that have shown surprising findings in their distress levels in relation to surgery types underscore the importance of a strong doctor-patient relationship to explain the findings [65, 66]. Patients who felt like they were able to collaborate and make well-informed informed decision about the type of surgery had significantly better long-term emotional adjustment [67, 68]. Over the past years, efforts have been undertaken to improve patient education by discussing these expectations extensively in the preoperative phase at our center, which may suggest that adequate patient-centered care may have had an influence on the patient wellbeing and functioning post-surgery [59, 68]. Since treatment expectancies or patient-care practices were not within the scope of this study, future studies should explore these further to test our assumptions and explore their relationship to quality of life in greater detail.

It was also found that women with no partner or children showed the most significant decline in psychosocial wellbeing and stable levels of emotional functioning. These findings corroborate with previous studies that highlight the importance of social support in the navigation and management of different phases of the cancer journey [12, 25–27]. While the other types of family situations did not significantly influence the psychosocial wellbeing or emotional functioning scores, not having any family may be reflective of the lack of flexibility in sharing the burden of cancer that is normally present and available to patients with an existing family system [69–72].

No significant effect of employment status or age at the time of diagnosis was found on the trends, which differed from the past studies [14, 15, 26, 27]. Breast cancer and chemotherapy have been associated with impaired physical functioning, leading to reduced work hours, exhaustion, cognitive declines and unemployment in about 10–20% of breast cancer patients [73, 74]. It is more than likely that some women after their diagnosis and following treatment may not go back to work, but this information is not recorded in Zorgmonitor, since the employment status was only recorded at T0. It is also important to consider differences in age between the employed and unemployed groups in our sample. The employed women were considerably younger than the unemployed women. Research has found that while the decision to return to work in half the breast cancer survivors was influenced by the cancer-related and familiar financial stress and strain [75, 76], and since age seems to have a moderating effect on the relationship between our outcome variables and employment, we may have had different results if we considered the interaction between age and work.

Past studies have found that younger women show greater amounts of distress through the cancer journey [57–65, 77–79]. However, just like with employment status, not controlling age-correlated variables like employment status, type of surgery and education level may have confounded our results. For instance, the group that underwent reconstruction in our study were younger than those who underwent mastectomy or lumpectomy. Since reconstruction is known to improve body-image and self-image compared to other types of surgery [80], it can be assumed that if the type of surgery was controlled for, there may have been an effect of age on psychological well-being. The average age of our participants being 52.7, which is quite young, in comparison to the average age of diagnosis in the Dutch population which is 61 [81], might have especially had an impact on not just the trends, but also on decisions related to age-controlled variables in question, namely type of surgery and employment status.

### Strengths and limitations

The strength of this study lies in its prospective character. Several past studies were retrospective in nature and collected data by asking the participants to look back on their journey, thereby possibly confounding the results with recall bias [5, 14, 25]. A sample size of 334 participants also provides the findings of this study with a good external validity [82]. Given that the baseline values found in this study for both the outcome variables are in line with the preoperative reference values in breast cancer and general population, we also take into consideration the minimally important difference (MID) for determining the clinical relevance of this study. MID, which is the smallest score difference on a health-related quality-of-life measure which makes it clinically relevant to the healthcare professionals, has been agreed to be 4 points for BREAST-Q and 10 points for EORTC-QLQ-C30 [83, 84]. Thus, given that the trajectories found in this study for emotional functioning and psychosocial wellbeing are in-line with these expectations, this study makes a clinically relevant contribution to the quality-of-life breast cancer literature.

A shortcoming of this study was the inconsistency in questionnaire administration. Because the BREAST-Q was not administered at T24, in order to understand how psychosocial wellbeing changed after a year, the trajectory had to be modeled (see Fig. 2). Data quality and value of these findings could have been enhanced if both questionnaires had been administered at the same time-points. When considering our analyses, this study poses a risk of Type 1 error due to the large number of analyses performed [82]. Due to the breast-specific questions within BREAST-Q, the choice of BREAST-Q to measure psychosocial wellbeing might have resulted in findings that are only reflective of breast-specific wellbeing and not wellbeing in a broader sense. While this was in-line with our operationalization of psychosocial wellbeing, this study would have benefitted with the aid of a general measure of wellbeing, such as PANAS or DASS next to BREAST-Q [7, 10].

Another limitation of the present study was that we did not control for whether women underwent systemic treatment. Systemic treatment may have an influence on the emotional and psychological outcomes of the patients, and therefore, it is recommended that future research includes this factor when exploring emotional functioning and psychosocial wellbeing in breast cancer survivors [56, 72]. As we noted in the discussion, many of the demographic and treatment characteristics might be (theoretically) related to one another, especially age. Therefore, if this research was to be replicated, controlling for age might yield a different essence to the findings. Further, the predictors we chose for this study were static. However, a lot of dynamic factors not only relate to the emotional and psychological adjustments, but also interact with the static factors to influence well-being outcomes in breast cancer survivors. Some of the dynamic factors we highlighted in the discussion were needs and individual expectations of the participants regarding their surgery. Additionally, instead of using demographic information about family status as proxy for social support, the women’s own appraisal of what constitutes social support and their coping strategies might be important in yielding a more complete profile of breast cancer patients who can be targeted for extra psychological support and more consistent screening [28, 56, 61].

## Conclusion

### Clinical implications

In general, it can be concluded from our study that women who elect a breast reconstructive surgery as opposed to mastectomy or lumpectomy tended to have better health-related quality of life outcomes, even though the impact of other demographic variables is less clear. One of the most significant decisions the patient makes in this phase concerns the type of surgery [55, 56]. Hence, our findings inform the need to foster early open dialogue between the patient and the professional about her preferences, expectations and the level of freewill in selecting a type of surgery [67, 85]. While most women in this sample underwent breast-conserving therapy, reflecting national data, this option is not feasible for some patients for example in large, multicentric carcinoma, DCIS, or after previous radiation treatment [64, 86]. If that were the case, and patients would have preferred this treatment option, patients should be given space and enough time to express disappointments, ask questions about other possibilities and process any loss of control they may feel over their decision. Breast reconstruction, on the other hand, is often a product of choice [26, 66, 67, 86]. However, the experience of women undergoing reconstruction may vastly differ based on the timing of reconstruction after mastectomy, type of reconstruction, and the occurrence of surgery-related complications [11, 26, 63, 64]. Hence, apart from expectations, these factors must also be discussed in depth before a decision is made.

This study also emphasizes the advantages of consistent use of PROMs in understanding patient’s health-related outcomes, and giving special attention to the deviations in scores in wellbeing at critical points in her trajectory, such as during diagnosis and within the pretreatment phase, where the woman learns of a seemingly traumatic change in her life and begins to learn how to make cancer a normal part of her life trajectory [20]. Providing hope and reassurance to the women even at the start that while it might seem like a very emotionally daunting and slow process, they, much like majority of the cancer survivors, will be able to return to their normal functioning eventually [12, 13]. This could give these women the opportunity to gather the informational, social and emotional resources they need in preparation for the potential points in their trajectory where their emotional functioning and psychosocial wellbeing are confronted by the most dysfunctions [12, 13, 23, 60, 69].

## Data Availability

The data that were analyzed during this study are available upon reasonable request from the corresponding author. The data are not publicly available due to privacy and ethical restrictions.

## Appendix

**Table:** **Table 3** Multi-level models

	Intercept			Linear time			Log time		
	Estimate	S.E.	*P*	Estimate	S.E.	*P*	Estimate	S.E.	*P*
**Emotional Functioning**									
**Main**	72.99	1.26	<.001	−0.04	0.14	.801	2.83	0.99	.004
** Type of surgery**									
BCT^a^	73.87	1.57	<.001	−0.06	0.17	.726	3.48	1.20	.004
Mastectomy^b^	−0.67	2.99	.822	0.32	0.38	.389	−4.20	2.47	.090
Reconstruction^b^	−6.88	4.14	.097	−0.49	0.46	.287	4.17	3.17	.189
** Family status**									
Partner & children^a^	70.51	1.82	<.001	−0.15	0.26	.562	3.55	1.67	.034
Partner^b^	5.06	2.91	.083	0.10	0.32	.304	−0.59	2.22	.791
Children^b^	5.20	6.27	.407	−0.21	0.98	.831	2.75	5.76	.634
No Partner/Children^b^	3.63	3.72	.329	0.74	0.65	.251	−5.14	3.76	.172
** Age**									
At mean age^a^	73.41	1.25	<.001	−0.03	0.14	.832	2.69	0.98	.006
Each year older^b^	0.41	0.10	<.001	−0.00	0.01	.980	−0.08	0.08	.336
** Employment status**									
Employed^a^	73.33	2.09	<.001	−0.24	0.28	.383	3.37	1.81	.063
Not employed^b^	−0.64	2.65	.808	0.27	0.33	.406	−0.55	2.17	.799
**Psychosocial wellbeing**									
**Main**	72.98	1.10	<.001	0.49	0.41	.237	−5.37	1.94	.006
** Type of surgery**									
BCT^a^	74.75	1.36	<.001	0.38	0.63	.543	−4.49	2.91	.123
Mastectomy^b^	−6.21	2.56	.016	0.20	0.92	.825	−1.77	4.32	.682
Reconstruction^b^	−1.54	3.51	.662	−0.14	1.10	.896	1.41	5.28	.790
** Family status**									
Partner & children^a^	70.53	1.59	<.001	0.64	0.59	.278	−5.54	2.78	.047
Partner^b^	4.84	2.51	.055	−0.61	0.91	.507	1.84	4.31	.670
Children^b^	2.26	5.46	.414	−0.48	1.80	.791	2.70	8.54	.753
No Partner/Children^b^	4.39	3.17	.166	0.63	1.34	.637	−4.46	6.22	.474
** Age**									
At mean age^a^	73.05	1.10	<.001	0.48	0.42	.260	−5.34	1.96	.007
Each year older^b^	0.08	0.08	.345	−0.01	0.03	.792	0.02	0.15	.902
** Employment status**									
Employed^a^	71.95	1.79	<.001	0.05	0.72	.947	−3.61	3.29	.273
Not employed^b^	1.45	2.27	.524	0.62	0.88	.484	−2.41	4.09	.555

*S.E* Standard error

^a^ reference

^b^ additional
